# Doctors in the Witness-Box

**Published:** 1921-07-16

**Authors:** 


### The Hospital

The Workers' Newspaper of Administrative Medicine and Institutional Life, Administration, National Insurance and Health.

No. 1832, Vol. LXX. SATURDAY, JULY 16, 1921. PRICE TWOPENCE

### DOCTORS IN THE WITNESS-BOX.

We make no apology for returning again to this important subject, which recent cases in the courts have brought prominently before the public, though there is nothing new in the question itself. It will, however, have come as a shock to many people to learn that no doctor can in law refuse to answer in the witness-box any question relating to his patient which in the opinion of the court may be relevant to the issue which is being tried. No matter how confidential the communications made by the patient may have been, the doctor is bound to disclose them or go to prison if he does not. We do not hesitate to say that if, as appears to be the case, this is the law, action is urgently required to put it right. The lawyer has from time immemorial enjoyed the privilege of refusing to disclose his client's secrets; and we cannot recollect a case, at any rate in recent times, in which any judge has dared even to threaten to commit a priest who has declined to divulge information which he has acquired in the exercise of his sacred office. A doctor is surely entitled to no less protection. He is the lay confessor of the community, and confidences made to him ought to be as sacred as those made under the seal of the confessional. The lawyer enjoys his privilege, we are told, for reasons of public policy; but the number of persons who are compelled to consult a lawyer in any one year is trifling compared to those who consult their doctor, and if public policy is admitted as a reason for the privilege in the one case, *à fortiori* it should be admitted in the other. The lawyer guards our material, the priest our spiritual, the doctor our physical and moral, interests. Whose function is the more important of the three, from the point of view of the community at large? He would be a bold man who argued that that of the lawyer so transcends those of the other two that he is justified in claiming for himself alone a privilege which he denies to them.

On the broadest grounds the case for the doctor is, in our opinion, unanswerable; but there are severely practical reasons at the present time which are not less strong. In a case before the Divorce Court the other day, Dr. Elliott, a well-known Cheshire doctor and the chief medical officer of the venereal disease clinic at Chester, which serves a large and populous district, was subpœnaed to give evidence relating to a patient who had been under his charge at the clinic. He objected that the information he was asked to disclose had been acquired by him as his patient's doctor, and that the treatment which she had been undergoing had been given under a pledge of secrecy. The judge, somewhat brusquely, told Dr. Elliott that none of these matters were relevant, and that he must answer the questions put. On which side, in such a case, do the greater interests of the community lie? On the one side we have the whole success of the campaign against venereal disease, the honour of the medical profession, the confidence of patients in their medical advisers; on the other, a litigation between two private individuals, of no interest to anyone but themselves. We do not wish to minimise the demands which the law makes upon all good citizens, and we recognise that it is entitled, in the interests of justice, to require that all information reasonably available shall be at its disposal for the sublime purpose of ascertaining the truth in every case, great or small. But it makes that demand in the interest of the body politic as a whole, and, as in the case of its own profession, it recognises that there may be limitations to it. Both justice and common sense require that those limitations should be extended further to meet the case of the medical profession.

A more difficult question no doubt arises in connection with the evidence of doctors in criminal cases. We doubt whether the argument on behalf of the doctors can be pressed so far as to include cases of this kind. There is a very real difference between litigation between private citizens and proceedings in which the Crown, that is to say the State in its corporate capacity, is prosecutor. The public policy which the doctor invokes for his protection in the one case may well be invoked against him in the other. It is, perhaps, desirable to suspend final judgment on this aspect of the matter; and, indeed, it is one which must be settled by public opinion in the last resort. It is not clear that public opinion would be prepared to allow crime to go unpunished for the sake of the Hippocratic oath; and doctors, who are practical men, will no doubt remember the homely proverb that half a loaf is better than no bread.

